# Supplementary material for: Multicenter Translational Trial of Remote Ischemic Conditioning in Acute Ischemic Stroke (TRICS BASIC)
Source: Stroke. 2025 Sep 26;56(12):3342–51. doi: 10.1161/STROKEAHA.125.051532 (PMC12643561; doi:10.1161/STROKEAHA.125.051532)
Supplement: Supplementary file 1 [file str-56-3342-s001.pdf]

Multi-center Translational Trial of Remote Ischemic Conditioning in Acute Ischemic Stroke (TRICS BASIC)

Simone Beretta*, MD, PhD, Mauro Tettamanti, PhD, Jacopo Mariani, PhD, Susanna Diamanti, MD, PhD, Alessia Valente, PhD, Ornella Cuomo, PhD, Chiara Di Santo, PhD, Ilaria Dettori, PhD, MartinaVenturini, PhD, Irene Bulli, PhD, Elisabetta Coppi, PhD, Manuel Alejandro Montano Castillo, PhD, Erica Butti, PhD, Giorgia Serena Gullotta, PhD, Martina Viganò, MD, Francesco Santangelo, MD, Francesco Andrea Pedrazzini, MD, Carlo Perego, PhD, Serena Seminara, PhD, Serenella Anzilotti, PhD, Joanna Rzemieniec, PhD, Laura Castiglioni, PhD, Benedetta Mercuriali, PhD, Majeda Muluhie, PhD, Chiara Paola Zoia, PhD, Gessica Sala, PhD, Luigi Sironi, PhD, Marco Bacigaluppi, MD, PhD, Gianvito Martino, MD, PhD, Felicita Pedata, PhD, Anna Maria Pugliese, PhD, Diana Amantea, PhD, Giacinto Bagetta, PhD, Antonio Vinciguerra, PhD, Giuseppe Pignataro, PhD, Stefano Fumagalli, PhD, Maria-Grazia De Simoni, PhD, Carlo Ferrarese, MD, PhD

Laboratory of Experimental Stroke Research, Department of Medicine and Surgery, University of Milano-Bicocca, Monza, Italy (S.B., J.M., S.D., M.V., F.S., F.A.P., C.F.). Department of Neurology and Stroke Unit, Fondazione IRCCS San Gerardo dei Tintori, Monza, Italy (S.B., C.F.). Milan Center for Neuroscience (NeuroMI), University of Milano-Bicocca, Milano, Italy (S.B., C.F.). Dipartmento di Politiche per la Salute, Istituto di Ricerche Farmacologiche Mario Negri IRCCS, Milano, Italy (M.T.). Dipartimento di Danno Cerebrale e Cardiovascolare Acuto, Istituto di Ricerche Farmacologiche Mario Negri IRCCS, Milano, Italy (A.V., S.F., M.D.S.). Division of Pharmacology, Department of Neuroscience, School of Medicine, University of Naples "Federico II", Naples, Italy (O.C., G.P., A.V.). Dipartimento di Farmacia e Scienze della Salute e della Nutrizione, Università della Calabria, Italy (C.D.S., D.A., G.B.). Department of Neuroscience, Psychology, Drug Research and Child Health (NEUROFARBA), University of Florence, Florence, Italy (I.D., M.V., I.B., E.C., F.P., A.M.P.). Neuroimmunology Unit, Institute of Experimental Neurology, IRCCS Ospedale San Raffaele, Milano, Italy (M.A.M.C., E.B., G.S.G., M.B., G.M.). Università Vita-Salute San Raffaele, Milano, Italy (M.A.M.C., E.B., G.S.G., M.B., G.M.). Department of Human Sciences and Quality of Life Promotion, San Raffaele University, Rome, Italy (S.A.). Dipartimento di Scienze Farmaceutiche, Università degli Studi di Milano, Italy (J.R., L.C., B.M., M.M., L.S.). Laboratory of Neurobiology, Department of Medicine and Surgery, University of Milano-Bicocca, Italy (C.P.Z., G.S.). Dipartimento di Scienze Biomediche e Sanità Pubblica, Università Politecnica delle Marche, Ancona, Italy (A.V.).

*Corresponding author. Email: simone.beretta@unimib.it

SUPPLEMENTAL MATERIAL

- **Figure S1:** Functional neuroscore of animals randomized to sham middle cerebral artery (MCA) surgery.
- **Figure S2:** Functional neuroscore of enrolled animals by single laboratories.
- **ARRIVE** Guidelines checklist.
- **Major Resources Table**

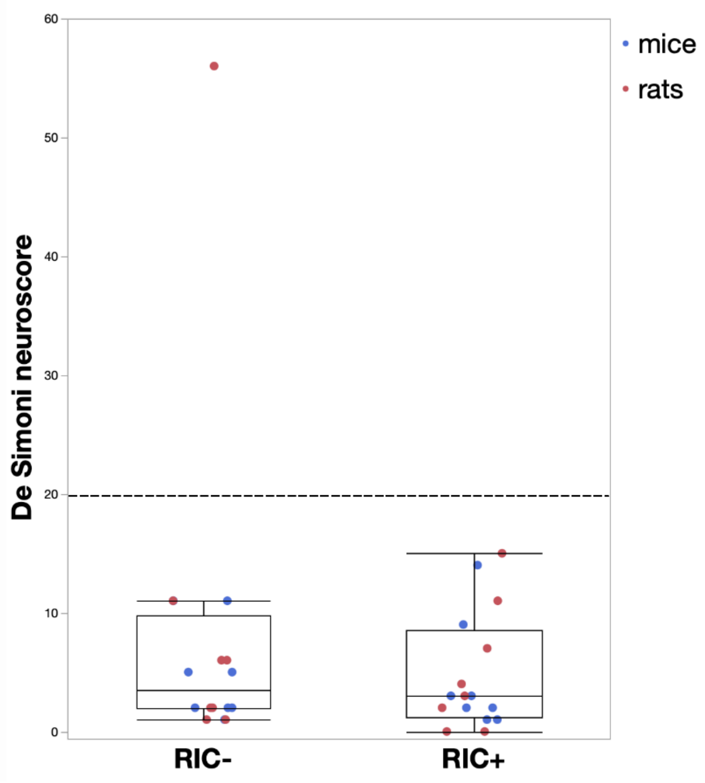

Fig. S1. **Functional neuroscore of animals randomized to sham middle cerebral artery (MCA) surgery.** Distribution of De Simoni neuroscore of individual animals treated with sham MCA surgery (mice in blue; rats in red). These animals were used as internal controls and were not included in the primary analysis. The threshold of 20 for "good functional outcome" is indicated with a dotted line. Data are expressed as box and whiskers plots (median, 25%-75% quartiles, minimum [25% quartile – 1.5*interquartile range], maximum [75% quartile + 1.5*interquartile range], with outliers).

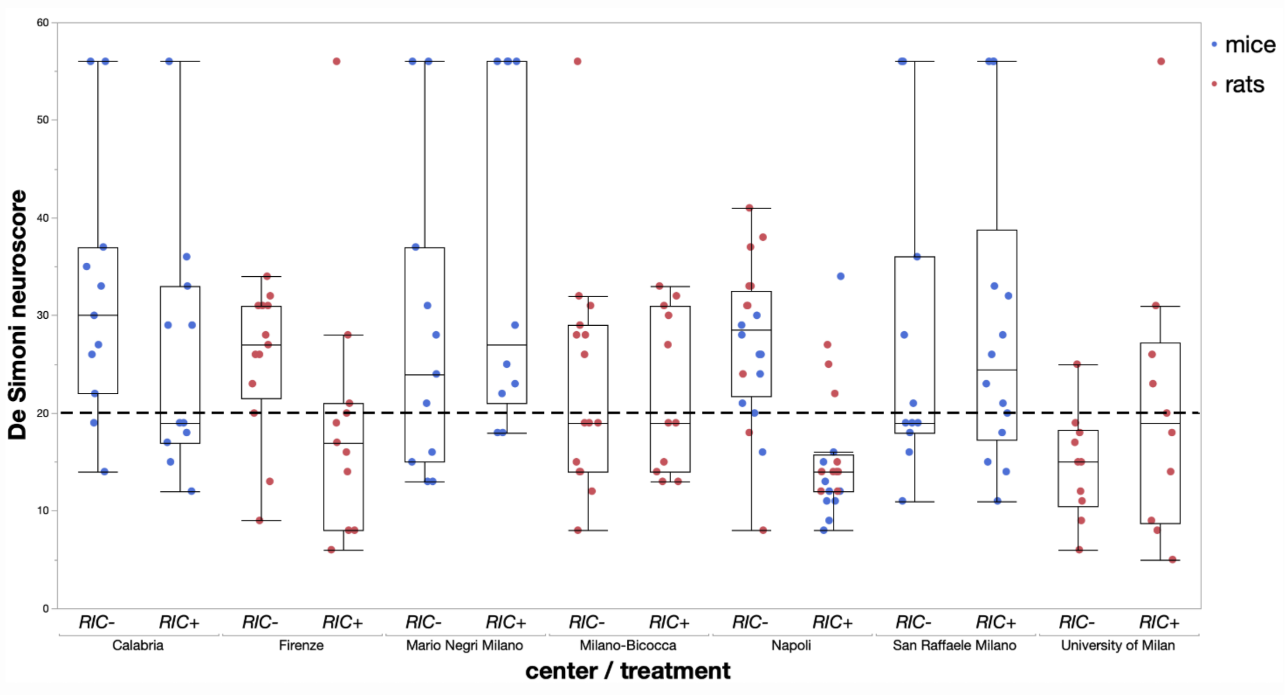

Fig. S2. Functional neuroscore of enrolled animals by single laboratories. Distribution of De Simoni neuroscore in mice (A) and rats (B) enrolled in the TRICS BASIC trial by single laboratories. The threshold of 20 for "good functional outcome" is indicated with a dotted line. Data are expressed as box and whiskers plots (median, 25%-75% quartiles, minimum [25% quartile – 1.5*interquartile range], maximum [75% quartile + 1.5*interquartile range], with outliers).

NOTE: Please save this file locally before filling in the table, DO NOT work on the file within your internet browser as changes will not be saved. Adobe Acrobat Reader (available free here) is recommended for completion.

The ARRIVE guidelines 2.0: author checklist

The ARRIVE Essential 10

These items are the basic minimum to include in a manuscript. Without this information, readers and reviewers cannot assess the reliability of the findings.

Item		Recommendation	Section/line number, or reason for not reporting
**Study design**	1	For each experiment, provide brief details of study design including:<br>a. The groups being compared, including control groups. If no control group has been used, the rationale should be stated.<br>b. The experimental unit (e.g. a single animal, litter, or cage of animals).	page 6, lines 24-26
**Sample size**	2	a. Specify the exact number of experimental units allocated to each group, and the total number in each experiment. Also indicate the total number of animals used.<br>b. Explain how the sample size was decided. Provide details of any *a priori* sample size calculation, if done.	page 7, 8-16
**Inclusion and exclusion criteria**	3	a. Describe any criteria used for including and excluding animals (or experimental units) during the experiment, and data points during the analysis. Specify if these criteria were established *a priori*. If no criteria were set, state this explicitly.<br>b. For each experimental group, report any animals, experimental units or data points not included in the analysis and explain why. If there were no exclusions, state so.<br>c. For each analysis, report the exact value of *n* in each experimental group.	page 8, 12-17<br>suppl. material
**Randomisation**	4	a. State whether randomisation was used to allocate experimental units to control and treatment groups. If done, provide the method used to generate the randomisation sequence.<br>b. Describe the strategy used to minimise potential confounders such as the order of treatments and measurements, or animal/cage location. If confounders were not controlled, state this explicitly.	page 7, 18-24
**Blinding**	5	Describe who was aware of the group allocation at the different stages of the experiment (during the allocation, the conduct of the experiment, the outcome assessment, and the data analysis).	page 7, 18-24
**Outcome measures**	6	a. Clearly define all outcome measures assessed (e.g. cell death, molecular markers, or behavioural changes).<br>b. For hypothesis-testing studies, specify the primary outcome measure, i.e. the outcome measure that was used to determine the sample size.	page 7, 1-7
**Statistical methods**	7	a. Provide details of the statistical methods used for each analysis, including software used.<br>b. Describe any methods used to assess whether the data met the assumptions of the statistical approach, and what was done if the assumptions were not met.	page 11-12
**Experimental animals**	8	a. Provide species-appropriate details of the animals used, including species, strain and substrain, sex, age or developmental stage, and, if relevant, weight.<br>b. Provide further relevant information on the provenance of animals, health/immune status, genetic modification status, genotype, and any previous procedures.	page 8, 1-3
**Experimental procedures**	9	For each experimental group, including controls, describe the procedures in enough detail to allow others to replicate them, including:<br>a. What was done, how it was done and what was used.<br>b. When and how often.<br>c. Where (including detail of any acclimatisation periods).<br>d. Why (provide rationale for procedures).	page 8, 4-12
**Results**	10	For each experiment conducted, including independent replications, report:<br>a. Summary/descriptive statistics for each experimental group, with a measure of variability where applicable (e.g. mean and SD, or median and range).<br>b. If applicable, the effect size with a confidence interval.	page 12-14

The Recommended Set

These items complement the Essential 10 and add important context to the study. Reporting the items in both sets represents best practice.

Item		Recommendation	Section/line number, or reason for not reporting
**Abstract**	11	Provide an accurate summary of the research objectives, animal species, strain and sex, key methods, principal findings, and study conclusions.	page 3-4
**Background**	12	a. Include sufficient scientific background to understand the rationale and context for the study, and explain the experimental approach.<br>b. Explain how the animal species and model used address the scientific objectives and, where appropriate, the relevance to human biology.	page 5
**Objectives**	13	Clearly describe the research question, research objectives and, where appropriate, specific hypotheses being tested.	page 6, 1-4
**Ethical statement**	14	Provide the name of the ethical review committee or equivalent that has approved the use of animals in this study, and any relevant licence or protocol numbers (if applicable). If ethical approval was not sought or granted, provide a justification.	page 6, 8-22
**Housing and husbandry**	15	Provide details of housing and husbandry conditions, including any environmental enrichment.	suppl. material
**Animal care and monitoring**	16	a. Describe any interventions or steps taken in the experimental protocols to reduce pain, suffering and distress.<br>b. Report any expected or unexpected adverse events.<br>c. Describe the humane endpoints established for the study, the signs that were monitored and the frequency of monitoring. If the study did not have humane endpoints, state this.	suppl. material
**Interpretation/ scientific implications**	17	a. Interpret the results, taking into account the study objectives and hypotheses, current theory and other relevant studies in the literature.<br>b. Comment on the study limitations including potential sources of bias, limitations of the animal model, and imprecision associated with the results.	page 15-17
**Generalisability/ translation**	18	Comment on whether, and how, the findings of this study are likely to generalise to other species or experimental conditions, including any relevance to human biology (where appropriate).	page 16, 11-24; page 17
**Protocol registration**	19	Provide a statement indicating whether a protocol (including the research question, key design features, and analysis plan) was prepared before the study, and if and where this protocol was registered.	page 4, line 5<br>page 6, line 9
**Data access**	20	Provide a statement describing if and where study data are available.	page 6, 11-13
**Declaration of interests**	21	a. Declare any potential conflicts of interest, including financial and non-financial. If none exist, this should be stated.<br>b. List all funding sources (including grant identifier) and the role of the funder(s) in the design, analysis and reporting of the study.	page 18, 9-10

Major Resources Table

In order to allow validation and replication of experiments, all essential research materials listed in the Methods should be included in the Major Resources Table below. Authors are encouraged to use public repositories for protocols, data, code, and other materials and provide persistent identifiers and/or links to repositories when available. Authors may add or delete rows as needed.

Animals (in vivo studies)

Species	Vendor or Source	Background Strain	Sex	Persistent ID / URL
Rats	Charles River	Sprague-Dawley	males and females	https://www.criver.com
Mice	Charles River	C57BL/6J	males and females	https://www.criver.com

Data & Code Availability

Description	Source / Repository	Persistent ID / URL
The data that support the findings of this study are available from the corresponding author upon reasonable request.		

ARRIVE GUIDELINES

The ARRIVE guidelines (https://arriveguidelines.org/) are a checklist of recommendations to improve the reporting of research involving animals. Key elements of the study design should be included below to better enable readers to scrutinize the research adequately, evaluate its methodological rigor, and reproduce the methods or findings.

Study Design

Groups	Sex	Age	Number (prior to experiment)	Number (after termination)	Littermates	Other description
Group 1 (RIC-)	males and females	Rats 8-9 weeks<br>Mice 10-12 weeks	43 mice<br>48 rats	39 mice<br>45 rats	Yes (prior to surgery)	4 mice excluded: no ischemic lesion at histology<br>3 rats excluded: no ischemic lesion at histology
Group 2 (RIC+)	males and females	Rats 8-9 weeks<br>Mice 10-12 weeks	45 mice<br>42 rats	43 mice<br>37 rats	Yes (prior to surgery)	2 mice excluded: no ischemic lesion at histology<br>5 rats excluded: no ischemic lesion at histology

Sample Size: A 20% rate of good functional outcomes was used as the baseline for animals subjected to MCA occlusion without effective treatment. An improvement of at least 30% (from 20% to 50%) was established as the minimum effect size of translational significance. Statistical significance (α) was set at 0.050 (two-tailed). Using a chi-squared test for data analysis, a total of 80 animals equally randomized between RIC+ and RIC- groups yielded a statistical power of 82%. The same calculation was applied to both species, requiring 160 animals to undergo MCA occlusion.

Inclusion Criteria: All rats which were subjected to successful MCA occlusion were included, judged as filament correctly positioned in the MCA origin during surgery AND a positive intra-ischaemic clinical assessment score. Animals will be judged ischaemic, and included in the trial, if presenting ≥3 of the following deficits after filament insertion:
1. The palpebral fissure has an ellipsoidal shape (not the normal circular one).
2. One or both ears extend laterally.
3. Asymmetric body bending on the ischaemic side.
4. Limbs extend laterally and do not align to the body.

Exclusion Criteria: Animals which either scored below the prespecified threshold of 2 of the intra-ischemic clinical score (see above) or with no ischemic lesion detected at brain histology were excluded from the analyses (i.e. animals without

stroke). Animals that died before RIC randomization were excluded (i.e.: animals not reaching the target randomization) and replaced by other animals, up to three per center. Conversely, animals that died after RIC randomization were retained in the prespecified (intention-to-treat) primary analyses and given the worst score: this was true for naturally dying animals and for animals sacrificed after showing signs of extreme distress.

Randomization: Two randomisation lists were produced, separately by species and stratified by centres and sex. The lists were produced using a pseudo-random number generator, using permuted blocks of random size. There were two successive randomizations: (1) allocation to receive an occlusion of the middle cerebral artery (MCA+) or its sham equivalent (MCA-), with a 5:1 ratio, and (2) allocation to receive a remote ischemic conditioning (RIC+) via transient femoral artery occlusion, or its sham equivalent (RIC-), with a 1:1 ratio.

Blinding: Functional and clinical assessments were conducted locally by researchers blinded to MCA occlusion and RIC treatment allocation, i.e. by a person not participating in/assisting the surgical practices. Centrally conducted histological procedures and data analysis (RIC+ vs RIC-) were conducted blinded to the group allocation.

DOI [to be added]
